# Injectable Buprenorphine for Self-Harming Behaviour: A Case Report

**DOI:** 10.1192/bjo.2025.10785

**Published:** 2025-06-20

**Authors:** Divya Vikraman Chandrika, Timothy Dye, Arif Alam

**Affiliations:** Cwm Taf Morgannwg University Health Board, South Wales, United Kingdom

## Abstract

**Aims:** Long-acting buprenorphine has been explored in context of its use in opioid dependence, however its potential for managing self-harm behaviour is limited. Self-harming behaviour often signals extreme emotional distress. This case report brings an insight into the effectiveness of injectable buprenorphine in a person with extreme self-harm behaviour.

**Methods:** Miss SD, 30-year-old single, unemployed woman, with history of emotional abuse, sexually abused by father from the age 6–8 and mother having difficulties with alcohol abuse. She has been involved with Psychiatric services from the age of 13 and had several Psychiatric hospital admissions, transfer to low secure Forensic unit and step down to supported accommodation. Having difficulties with self-harming behaviours, suicidal thoughts or attempts and aggression towards staff. Self-harm behaviour included cutting, overdoses, ligatures; to an extent she needed multiple surgical interventions for cutting, inserting glass and glueing the genitalia multiple times, along with glueing nose and lips.

Over the course her diagnoses included EUPD, PTSD, Depressive disorder, transient Psychotic episodes, alcohol dependence and polysubstance misuse.

Due to escalating behaviour she was trialled on injectable buprenorphine as off-licence use for self-harming behaviour. We have compared the data of 2 years on injectable buprenorphine to history prior to that.

Initiation on buprenorphine treatment had a dramatic improvement in Miss SD given the number of incidents of both alcohol use and self-harm.

**Results:** Buprenorphine treatment had a greater reduction in self-harming behaviour in Miss SD. There have been only 1–2 episodes of self-harm per year since she started on buprenorphine i.e, in last 2 years, when compared with the several episodes a week. In addition the number of hospital admissions reduced to 2 brief admissions, compared with several admissions including long stay in Low Secure Unit.

Recurrent self-harm can have an endogenous opioid response, and in the long term the pattern is similar to addiction.

Buprenorphine is a partial μ-opioid receptor agonist and a potent kappa antagonist, and treatment with long-acting opioid antagonist could block the reward of enhanced endogenous opioids caused by self-harming behaviours and subsequently lead to their extinction.

**Conclusion:** A retrospective case study of Miss SD since the initiation of buprenorphine suggests this may be an alternative therapeutic option to treat people with significant Self-harming behaviour with functional impairment. In addition it is much safer as it causes less respiratory depression than other opioids.

The evidence is promising, however more research is needed to review the effectiveness, establish efficacy, and safety.

